# Tetanus. Chloroform

**Published:** 1854-12

**Authors:** 


TETANUS. CHLOROFORM.—Encouraged by the effects of chloroform in two cases of tetanus recorded by Drs. V. Dusche and Langenbecks, Dr. Panthel employed it in a severe traumatic case in a strong man, aged twenty, who was first treated by bleeding and opium, then by chloroform. The relief given by the inhalation of chloroform (not carried to unconsciousness) was extraordinary, so that the patient could take food, yet in spite of it the spasms returned as severely as if no chloroform had been used, although they were invariably removed when the inhalation was again resorted to; the patient died two days after it was commenced. During the first day of this treatment no less than 6 ounces of chloroform were used, with invariable benefit, and its use was continued till the fatal termination. Although the chloroform was thus unable to save life, the relief it gave to the pain was an immense benefit.—*Henle's Zeitschrift.*

